# No surgery, but headache exists? Quality of life with vestibular schwannoma

**DOI:** 10.1007/s00405-026-10120-3

**Published:** 2026-03-09

**Authors:** Ivan Stoyanov, Marian Radev, Miriam Simon, Jan Frederick Cornelius, Jörg Schipper, Julia Kristin

**Affiliations:** 1 Department of Otorhinolaryngology, Düsseldorf University Hospital, Moorenstrasse 5, Düsseldorf, 40225 Germany; 2 Department of Neurosurgery, Düsseldorf University Hospital, Moorenstrasse 5, Düsseldorf, 40225 Germany

**Keywords:** Vestibular Schwannoma (VS), Health, Related Quality of Life (HRQoL), Pain Assessment, Non, Operated Patients, Penn Acoustic Neuroma Quality, Of, Life Scale (PANQOL), Short, Form McGill Pain Questionnaire (SF, MPQ), Minimal Clinically Important Difference (MCID)

## Abstract

**Objective:**

The aim of this study was to analyze self-reported pain symptoms on the side of the vestibular schwannoma (VS) and their impact on health-related quality of life (HRQoL) in conservatively managed VS- patients.

**Study design:**

Prospective clinical study.

**Setting:**

Department of Otorhinolaryngology, Department of Neurosurgery, Single-center University Hospital, Certified skull base center, Germany.

**Patients:**

A total of 163 conservatively managed patients at the time of initial diagnosis or during wait-and-scan strategy with radiologically confirmed unilateral VS. Exclusion criteria: prior surgery, radiotherapy, or bilateral tumors, as well as neurofibromatosis type II (NFII).

**Interventions:**

Assessment using the Penn Acoustic Neuroma Quality-of-Life Scale (PANQOL), and for a subset of patients (n = 80/163), the Short-Form McGill Pain Questionnaire (SF-MPQ).

**Main outcome measures:**

Prevalence of pain; differences in HRQoL scores; correlations between pain and age, tumor size, and psychosocial factors via t-test and ANOVA; assessment of clinical relevance using the Minimal Clinically Important Difference (MCID).

**Results:**

Overall, 48.75% of patients reported experiencing pain. Patients with pain showed a significantly reduced HRQoL in the domains of "anxiety", "energy", and the overall PANQOL score (each p < 0.0001). Patients under 58 years of age reported significantly more pain than those over 58 years (p = 0.000380). Tumor size, gender, educational or occupational status showed no significant correlations.

**Conclusion:**

Pain can significantly impair HRQoL even in conservatively managed VS patients. The cause of the reported pain remains unclear; psychosomatic factors may contribute to or modulate pain perception. It is however also possible, that reported pain is not solely attributable to the tumor itself, but could instead reflect underlying psychosocial factors such as heightened anxiety and reduced energy levels.

## Introduction

The primary symptom of vestibular schwannoma (VS) is unilateral hearing loss, reported in up to 90–95% of cases [1]. Other typical symptoms include vestibular deficits (80%), tinnitus, and coordination disorders [1, 2]. In rare and more advanced cases, headaches, nausea, and vomiting may occur [3]. In contrast, pain is a frequent accompanying symptom in other intracranial tumors such as meningiomas or gliomas. This pain can be caused by disturbances in cerebrospinal fluid flow, or neurovascular irritation [4].

Pain in VS may have a neuropathic origin (e.g., trigeminal involvement) [5], or a somatic cause such as dural irritation from large tumors [6]. VS-related pain can develop through different mechanisms. Neuropathic pain is often due to compression or stretching of the trigeminal nerve, potentially resulting in facial pain or even classic trigeminal neuralgia, in rare cases even in tumors of small size [5, 7, 8]. Somatic pain, on the other hand, typically results from dural traction or irritation caused by tumor growth into the cerebellopontine angle, which frequently manifests as occipital or generalized headache [9]. In some patients, a reduced sensitivity on the posterior wall of the external auditory canal can be observed, known as the "Hitselberger sign." This is caused by irritation of the nervus intermedius. Pain in the ear area can also be attributed to compression of the nervus intermedius [10].

However, pain cannot always be fully explained by anatomical or physiological mechanisms, as it is often disproportionate to objective clinical findings and is significantly influenced by psychological modulation [11–13]

Psychological factors such as anxiety, depression, and chronic stress play a key role in amplifying pain perception and chronicity. These factors are known to lower pain thresholds and increase subjective pain severity [11, 14, 15].

Additionally, persistent pain can affect mental health causing anger, frustration and depression—thus creating a bidirectional relationship in which distress both contributes to and results from chronic pain [13]. Conversely, chronic pain can significantly impair mental health and thus negatively impact quality of life [14].

It is known that despite their benign nature, VS can significantly affect health-related quality of life (HRQoL) [16–18]. Key therapeutic aims include the preservation of hearing and facial nerve function, along with the maintenance of a high quality of life related to the specific disease [19]. HRQoL assessment also aids in evaluating treatment outcomes and guiding therapeutic decision-making [18].

The aim of this prospective clinical study was to assess the perception of pain in conservatively managed patients with VS at the time of initial diagnosis or during wait-and-scan strategy using the PANQOL and SF-MPQ questionnaires and to examine its impact on health-related quality of life (HRQoL).

## Materials and methods

### Patient cohort

A total of n = 163 patients with radiologically confirmed unilateral VS were included. These patients presented either for the first time at our outpatient department or as part of a "wait-and-scan" strategy at the ENT or Neurosurgery Department of University Hospital Düsseldorf. Exclusion criteria were prior surgery, previous radiotherapy, or bilateral VS (suggestive of NF2). Data collection was conducted following informed consent and in accordance with a positive vote from the local ethics committee.

### Penn acoustic neuroma quality-of-life (PANQOL) questionnaire

To assess disease-specific quality of life, the German version of PANQOL questionnaire was used [20–22]. Demographic and clinical parameters (age, sex, occupation, education level, tumor size according to Koos classification) were also recorded.

### Short-form McGill pain questionnaire (SF-MPQ) questionnaire

For more detailed pain assessment, the Short-Form McGill Pain Questionnaire (SF-MPQ) was additionally used [23–25], which is a shortened version of the McGill Pain Questionnaire developed by Melzack et al. [26].

It includes 15 descriptors of pain (11 sensory, 4 affective), a visual analog scale (VAS), and a pain intensity scale to provide a comprehensive overview of the pain experience [26–29]. The combination of both questionnaires enables a more comprehensive evaluation of the pain impact.

### Description of statistical methods used

Initially, only the PANQOL data were analyzed. The Shapiro–Wilk test was used to test for normal distribution. Group comparisons were performed using t-tests and one- and two-way ANOVA, supplemented by post hoc tests such as Tukey HSD, Šídák, and Dunnett. Subsequently, pain results from the SF-MPQ were correlated with PANQOL data. For this, Section III of the SF-MPQ, representing overall subjective pain evaluation, and the "pain" domain of PANQOL were used. T-tests and ANOVA were again applied in this analysis.

## Results

A total of n = 163 conservatively managed patients with VS were included in the analysis (n = 91 initial consultations, n = 72 as part of a “wait-and-scan” strategy). The cohort comprised 75 women and 88 men, with a median age of 58 years (range: 18–84 years) examined in the period from January 2023 to December 2024. Tumor sizes according to the Koos classification were distributed as follows: Koos I n = 72, Koos II n = 54, Koos III n = 16, Koos IV n = 14. In n = 7 patients tumor size could not be assessed or could not be classified using the Koos system (e.g., intracochlear schwannomas). The majority of patients (n = 118) were employed in jobs without significant physical demands, while n = 29 had physically demanding occupations. An academic degree was held by 49 patients, while n = 80 had a non-academic educational background. Data on educational background was available for n = 129 patients; for the remaining patients, educational level was not specified or could not be determined retrospectively.

In the PANQOL “pain” domain, a significant age-related difference was observed: patients over 58 years of age had significantly higher HRQoL pain scores (80.77%) compared to those aged 58 or younger (HRQoL pain = 71.47%) (p = 0.0004).

No significant gender differences were found in PANQOL (p = 0.066): The mean HRQoL pain score was 72.67% for women and 80.40% for men. A total of n = 91 patients at initial diagnosis in our outpatient department and an additional n = 72 patients undergoing wait-and-scan therapy were analyzed using an unpaired t-test in order to determine whether the intensity of pain differs at the onset of the disease, shortly after the diagnosis is disclosed, compared to when the diagnosis is already known. The analysis revealed no significant difference in the HRQoL pain scores between the first visit group (74.73%) and the wait-and-scan group (79.35%).

A one-way ANOVA showed no significant association between tumor size (Koos I–IV) and reported pain symptoms (p = 0.212). This analysis was performed using all patients the with available Koos classification (n = 156).The Tukey-HSD post hoc test was used to identify any specific group differences, as ANOVA only indicates overall significance. It also revealed no significant differences between these tumor size groups.

Occupational status (p = 0.160) and educational level (p = 0.351) similarly showed no significant association with pain burden.

### Correlation between results in PANQOL “Pain” domain and the domains “anxiety,” “energy,” “general health,” and the overall PANQOL score

Patients (n = 163) were divided into two groups based on their response to PANQOL question 14 („I have problems with head pain on the side of my acoustic neuroma tumor”): “No Pain” (n = 117) included patients who responded with a 1 or 2 to the pain item in the PANQOL questionnaire (1 = “strongly disagree”, 2 = “disagree”), while the “Pain” group (n = 46) comprised those who answered with 3, 4, or 5 (3 = “neutral”, 4 = “agree”, 5 = “strongly agree”). In the German version of the questionnaire, response 3 is labeled “partly/partly” or “sometimes”, since this corresponds to the familiar distribution of 5 possible responses in questionnaires of this kind in German. It was interpreted as indicating that the patient experiences pain intermittently or occasionally. Therefore, those selecting option 3 were included in the “Pain” group.To ensure comparability, domain scores were normalized and converted to percentages (0–100%, with higher values indicating better HRQoL).

Patients reporting pain had significantly lower quality of life scores in the domains of “Anxiety” (74.2% vs. 52.2%; p < 0.0001), “Energy” (75.1% vs. 50.9%; p < 0.0001), and in the overall PANQOL score (77.2% vs. 56.1%; p < 0.0001).

There was no significant difference in the “General Health” domain (62.8% vs. 61.7%; p = 0.6905).

### Results of the SF-MPQ analysis and correlation with PANQOL

A total of n = 80 out of 163 patients completed both the PANQOL and SF-MPQ questionnaires. A comparison of pain intensity (SF-MPQ Section III) showed that patients aged ≤ 58 years reported significantly higher pain scores than those aged > 58 years (p = 0.0036). This finding was consistent with the PANQOL “Pain” domain results, in which younger patients also reported significantly lower HRQoL scores (p = 0.0185).

No significant differences were found with respect to gender (p = 0.3169), tumor size according to the Koos classification (p = 0.6801), or occupational/educational status (p > 0.05).

Patients who reported pain in the SF-MPQ had significantly reduced HRQoL in the PANQOL domains “Anxiety” (p < 0.0001), “Energy” (p = 0.0003), and the overall PANQOL score (p < 0.0001), compared to those who reported no pain in the SF-MPQ. No significant association was found in the “General Health” domain (p = 0.8118).

An analysis was conducted in order to determine how many of the n = 80 patients who completed both questionnaires reported experiencing pain. The results showed that 27.5% of patients indicated pain in the PANQOL questionnaire, whereas 48.75% reported pain in the SF-MPQ. The pain was mostly characterized as throbbing and stabbing. For 61.5% of those affected, it was rated as mild or discomforting, for 36% as distressing, and for 2.5% as excruciating. Using the SF-MPQ, we identified an additional 17 patients (21.25% of the total sample of n = 80) who did not report any pain in the PANQOL questionnaire. Among these 17 patients, 47% described their pain as mild, 35% as discomforting, and 18% as distressing.

**Fig. 1:**
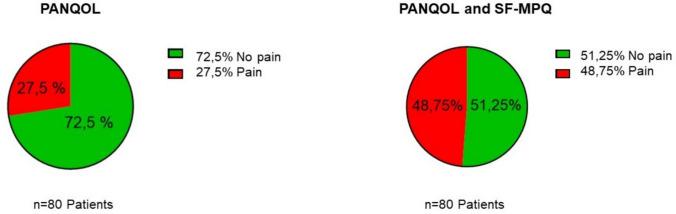
Comparison of self-reported pain prevalence in PANQOL versus SF-MPQ. The pie chart illustrates that 27.5% of patients reported pain in PANQOL, whereas 48.75% did so in SF-MPQ, demonstrating a discrepancy of over 21% between the instruments

This represents a difference of over 21%, highlighting a notable discrepancy between the two instruments in capturing patient-reported pain. Importantly, all patients who reported pain in the PANQOL also reported it in the SF-MPQ as well (see Fig. 1).

### MCID calculation for relevant results

For the PANQOL domains “Anxiety,” “Energy,” and the overall score, it was assessed whether the statistically significant differences between the pain and no-pain groups were clinically meaningful, based on the Minimal Clinically Important Difference (MCID) as defined by Carlson et al. [30]. All differences clearly exceeded the MCID threshold: Anxiety (Δ = 24.75), Energy (Δ = 24.77), Overall Score (Δ = 21.53). Therefore, the differences are considered clinically relevant (see Table 1).

**Table 1:** Comparison of PANQOL domain scores between patients reporting pain and those without pain. The minimum clinically important difference (MCID) thresholds are used according to the study by Carlson et al. [30], allowing for interpretation of whether group differences are clinically meaningful

**PANQOL-Domain**	**Mean** **(No Pain)**	**Mean** **(With Pain)**	**Difference (Δ)**	**MCID Threshold (per Carlson et al. **[30]***)***	**Clinically relevant**
Anxiety	76,92	52,17	24,75	11	Yes
Energy	75,68	50,91	24,77	13	Yes
Overall Score	76,73	55,20	21,53	11	Yes

## Discussion

This study provides new insights into the prevalence and clinical relevance of pain symptoms in conservatively managed patients with VS. Previous literature indicates that around 2–5% of VS patients report pain at initial diagnosis [2, 31]. By contrast, prospective surveys and long-term follow-ups reveal a much higher incidence of pain in VS patients. Carlson et al. reported that approximately half of VS patients (≈50%) experience headaches of varying severity about 8 years after diagnosis or treatment and that patients managed with observation were more than twice as likely to suffer from severe headache disability compared to controls [15].

This highlights pain as an often under-recognized yet prevalent symptom in VS, which requires closer attention even in “wait-and-scan” management. While most existing studies focus primarily on postoperative pain with up to 64% of patients reporting pain [1, 18, 19, 32–35], our data show that 48.75% of patients report pain at their first presentation in our outpatient department or during a “wait-and-scan” strategy, exceeding the global headache prevalence reported by the WHO in the general population (40%) [36]. This aligns with findings indicating that between one-third and nearly half of VS patients experience persistent headaches long after diagnosis or treatment—regardless of whether they had undergone surgery, radiosurgery, or conservative management [15, 37]. However, these figures are not directly comparable, as our data reflects self-reported tumor-associated pain symptoms rather than clinically diagnosed headache disorders.

Patients under 58 years of age reported significantly higher pain levels in both PANQOL and SF-MPQ (p < 0.01), consistent with earlier studies on postoperative pain in VS [37, 38]. Age-related differences in pain perception have been reported across both general and VS-specific populations. Different studies show that younger VS patients were more likely to report severe, persistent headaches [15, 35, 39].

No significant gender differences were found in our cohort, in contrast to other studies that have reported a higher self-reported pain prevalence in women [15, 37, 40, 41].

One finding of this study is that occupational and educational status do not significantly correlate with pain intensity. As these factors are rarely examined in VS-specific research, direct comparisons are not possible. However, evidence from broader pain literature suggests that lower education, unemployment, and other socioeconomic disadvantages are often associated with higher self-reported pain prevalence, greater severity, and increased disability. Some studies also indicate that job position may be a stronger predictor of pain intensity than education or income alone [42, 43].

Tumor size (Koos I–IV) showed no influence on reported pain, with the majority of tumors in our cohort being classified as Koos I and II. The number of patients with large VS in our cohort was small (Koos IV, n = 14; 8.6%), limiting statistical power and precluding reliable conclusions regarding size-related headache mechanisms. Therefore, these findings primarily apply to small, conservatively managed VS and should not be extrapolated to patients with larger tumors or to those undergoing surgical or radiotherapeutic treatment. Other studies have found that smaller tumors are associated with increased postoperative headache [37, 38, 44–46]. Our findings suggest that negative influencing factors (e.g., lack of energy, anxiety) may contribute to pain perception independently of tumor-related mechanical factors (e.g. tumor pressure).

Given the significant reduction in HRQoL in the PANQOL domains of anxiety and energy, psychosomatic complaints may be a contributing factor. The consideration of psychosomatic factors is based on our statistically and clinically relevant findings showing that patients reporting pain experience reduced HRQoL in the PANQOL domains of anxiety and energy. However, the absence of a significant difference in HRQoL pain scores that was observed between patients at first presentation and those managed under a wait-and-scan strategy does not support a purely psychosomatic explanation of pain in VS patients and suggests that additional mechanisms beyond an initial psychological stress response may contribute to pain perception. It is also possible that these psychological burdens contribute to or exacerbate the perception of pain.

Studies support a bidirectional relationship between pain and psychological symptoms. Pain may induce anxiety, helplessness, and fatigue, which in turn lower pain thresholds and increase distress [11] while psychological comorbidities are more closely linked to headache-related disability in VS patients than to tumor size or treatment modality [15]. Similarly, Pruijn et al. identified anxiety, fatigue, and headache as key predictors of reduced physical and mental HRQoL in VS patients [47].

Our results demonstrate a significant impairment in HRQoL among patients reporting pain compared to those without pain. This suggests that pain should not be viewed in isolation, but rather as being closely associated with psychological and functional impairments, as previously described in the literature [12, 28, 48, 49]. Importantly, these differences were not only statistically significant but also clinically meaningful, as they exceeded established MCID thresholds. This emphasizes the need for a multidisciplinary approach to VS care, with integration of psychosocial support and symptom-based management even in conservatively treated patients. Persistent pain and impaired HRQoL may be considered among the factors influencing a transition from a wait-and-scan approach to active treatment, for example surgery. Comparable shifts in therapeutic decision-making have been reported in the context of hearing preservation, where studies suggest potential benefits of earlier intervention in selected patients with small tumors [50, 51].

An interesting finding is the discrepancy between pain assessment using the PANQOL and the SF-MPQ. The SF-MPQ was administered in a subset of patients (n = 80), allowing a more detailed pain assessment. While the PANQOL includes only a single question related to pain, the SF-MPQ allows for a more thorough evaluation of both sensory and affective components of pain, focusing exclusively on this issue. In contrast, PANQOL captures a broad range of disease-specific symptoms. In our analysis, 27.5% of patients reported pain in PANQOL, whereas the SF-MPQ identified pain in 48.75% of cases. This means that an additional 21.25% of patients with pain were identified using the SF-MPQ, raising the question of whether PANQOL is sufficiently sensitive for pain assessment in VS patients and suggests that PANQOL may underestimate pain symptoms compared to dedicated pain instruments. A more comprehensive pain assessment may therefore be relevant not only for research purposes but also for clinical decision-making and patient counseling. Additional tools such as the Vestibular Schwannoma Quality of Life Index (VSQOL) questionnaire [52] may be employed in future studies to compare and further improve the detection of specific impact factors [53].

### Limitations

This study has several limitations. The PANQOL specifically asks: “I have problems with head pain on the side of my acoustic neuroma tumor.” Due to this explicit question, tension headaches or migraines are not expected to be reported, as far as the patient is capable of making a distinction. Despite the phrasing of the question, misinterpretation cannot be ruled out. Second, our classification of patients into the “pain” group included those who selected the response “teils/teils” (i.e., “partly/partly” or “sometimes”) in the German PANQOL version. While this was interpreted as an indication of intermittent pain, this categorization may have overestimated the actual number of patients experiencing clinically relevant pain. Third, most tumors in our cohort were Koos I and II, limiting our ability to assess whether tumor size truly has no influence on pain. Although no significant size-related correlation was found, this result may not apply to patients with larger tumors. However, patients with larger tumors (Koos III and IV) usually require active therapy thus the role of headache in conservative setting is for these patient groups less relevant. Our statistical results are based on univariate analyses. Given the exploratory nature of the study and the sample size all results should be interpreted as exploratory and hypothesis-generating rather than independent effects.

Finally, the study was conducted at a single university hospital, which may limit the generalizability of findings to broader VS populations, particularly those managed in non-academic or community settings. Furthermore, all questionnaires were administered in German language, therefore cultural or language-specific factors may affect symptom perception and reporting. For this reason, the generalizability of our findings may be limited to similar hospital settings and to conservatively managed VS populations within comparable healthcare systems.

### Implications and outlook

Our findings highlight the importance of systematic pain assessment even in conservatively managed VS patients. Interdisciplinary care strategies, including pain management and psychosocial support, should be considered.

Long-term follow-up studies are needed to better understand how changes in pain levels affect health-related quality of life (HRQoL) over time.

## Conclusion

This study demonstrates that pain can have a significant impact on HRQoL even in conservatively managed VS patients. Pain was found to be significantly associated with reduced quality of life. The SF-MPQ proved to be more sensitive than PANQOL in capturing pain symptoms. These findings highlight the potential relevance of psychosomatic factors and emphasize the need for interdisciplinary patient care with a particular focus on optimized pain management.

## Data Availability

Data are available from the corresponding author upon reasonable request.

## References

[1] Wilcke O (1973) Differentialdiagnose der Tumoren im Kleinhirnbrückenwinkel. Acta Neurochir 28(4):305–313 4543994 10.1007/BF01405648

[2] Foley R et al (2017) Signs and symptoms of acoustic neuroma at initial presentation: an exploratory analysis. Cureus. 10.7759/cureus.1846 29348989 10.7759/cureus.1846 PMC5768319

[3] Gupta VK, Thakker A, Gupta KK (2020) Vestibular schwannoma: what we know and where we are heading. Head Neck Pathol 14(4):1058–1066 32232723 10.1007/s12105-020-01155-x PMC7669921

[4] Evers S (2018) Kopfschmerzen bei Hirntumoren. Nervenheilkunde 37:53–58

[5] Neff BA et al (2017) Trigeminal neuralgia and neuropathy in large sporadic vestibular schwannomas. J Neurosurg 127(5):992–999 28084915 10.3171/2016.9.JNS16515

[6] Medicine, J.H. *Vestibular Schwannoma*. 03.03.2025]; Available from: https://www.hopkinsmedicine.org/health/conditions-and-diseases/brain-tumor/vestibular-schwannoma.

[7] Onoda K et al (2022) Small vestibular schwannoma presented with trigeminal neuralgia: illustrative case. J Neurosurg Case Lessons 4(9):Case22274 36051778 10.3171/CASE22274 PMC9426354

[8] Ananthan S, Kumar U, Johnson S (2024) A rare case of vestibular schwannoma manifesting as trigeminal neuralgia. J Am Dent Assoc 155(2):177–183 38032593 10.1016/j.adaj.2023.10.004

[9] Jackler RK, Pitts LH (1990) Acoustic neuroma. Neurosurg Clin N Am 1(1):199–223 2135969

[10] Hitselberger WE, House WF (1966) Acoustic neuroma diagnosis: external auditory canal hypesthesia as an early sign. Arch Otolaryngol Head Neck Surg 83(3):218–221 10.1001/archotol.1966.00760020220007 5904042

[11] Michaelides A, Zis P (2019) Depression, anxiety and acute pain: links and management challenges. Postgrad Med 131(7):438–444 31482756 10.1080/00325481.2019.1663705

[12] Eich W et al (2023) Psychosocial factors in pain and pain management: a statement. Schmerz 37(3):159–167 35303149 10.1007/s00482-022-00633-1 PMC10229715

[13] Hadi MA, McHugh GA, Closs SJ (2019) Impact of chronic pain on patients’ quality of life: a comparative mixed-methods study. J Patient Exp 6(2):133–141 31218259 10.1177/2374373518786013 PMC6558939

[14] Eich W et al (2023) Psychosoziale Faktoren bei Schmerz und Schmerzbehandlung. Schmerz 37(3):159–167 35303149 10.1007/s00482-022-00633-1 PMC10229715

[15] Carlson ML et al (2015) Risk factors and analysis of long-term headache in sporadic vestibular schwannoma: a multicenter cross-sectional study. J Neurosurg 123(5):1276–1286 26090830 10.3171/2014.12.JNS142109

[16] Gauden A et al (2011) Systematic review of quality of life in the management of vestibular schwannoma. J Clin Neurosci 18(12):1573–1584 22014598 10.1016/j.jocn.2011.05.009

[17] Scheich M et al (2024) Preoperative quality of life in patients with small vestibular schwannomas. J Int Adv Otol 20(6):472–476 39659231 10.5152/iao.2024.241481 PMC11639557

[18] Bender M, Tatagiba M, Gharabaghi A (2022) Quality of life after vestibular schwannoma surgery: a question of perspective. Front Oncol 11:770789 35223451 10.3389/fonc.2021.770789 PMC8873590

[19] Yildiz E et al (2022) Vestibular schwannoma: diagnosis-therapy-aftercare. Wien Med Wochenschr 172(1–2):2–7 33439379 10.1007/s10354-020-00800-y PMC8837524

[20] Kristin J et al (2019) Patient quality of life after vestibular schwannoma removal: possibilities and limits to measuring different domains of patients’ wellbeing. Eur Arch Otorhinolaryngol 276(9):2441–2447 31177326 10.1007/s00405-019-05499-1

[21] Glaas MF et al (2018) Quality of life after translabyrinthine vestibular schwannoma resection-reliability of the German PANQOL Questionnaire. Otol Neurotol 39(6):e481–e488 29889791 10.1097/MAO.0000000000001819

[22] Kristin J et al (2017) Multistep translation and cultural adaptation of the Penn acoustic neuroma quality-of-life scale for German-speaking patients. Acta Neurochir (Wien) 159(11):2161–2168 28861705 10.1007/s00701-017-3304-z

[23] Melzack R (1975) The McGill pain questionnaire: major properties and scoring methods. Pain 1(3):277–299 1235985 10.1016/0304-3959(75)90044-5

[24] Stein C, Mendl G (1988) The German counterpart to McGill Pain Questionnaire. Pain 32(2):251–255 3362561 10.1016/0304-3959(88)90074-7

[25] Kiss I, Müller H, Abel M (1987) The McGill pain questionnaire — german version. A study on cancer pain. Pain 29(2):195–207 2886967 10.1016/0304-3959(87)91036-0

[26] Melzack R (1987) The short-form McGill pain questionnaire. Pain 30(2):191–197 3670870 10.1016/0304-3959(87)91074-8

[27] Niederhagen B et al (1997) Postoperative pain after interventions in the area of the mouth-jaw-face. Mund Kiefer Gesichtschir 1(4):229–234 9410633 10.1007/BF03043555

[28] Brünahl CA et al (2014) Psychosomatic aspects of chronic pelvic pain syndrome. Psychometric results from the pilot phase of an interdisciplinary outpatient clinic. Schmerz 28(3):311–8 24728530 10.1007/s00482-014-1422-6

[29] Lang PM et al (2009) Correlation between quantitative sensory testing and questionnaires on neuropathic pain for chronic ischemic pain in peripheral arterial disease. Schmerz 23(3):251–4 19280230 10.1007/s00482-009-0773-x

[30] Carlson ML et al (2015) The minimal clinically important difference in vestibular schwannoma quality-of-life assessment: An important step beyond P <.05. Otolaryngol Head Neck Surg 153(2):202–8 26038393 10.1177/0194599815585508

[31] Fernández-Méndez R et al (2023) Incidence and presentation of vestibular schwannoma: a 3-year cohort registry study. Acta Neurochir (Wien) 165(10):2903–2911 37452904 10.1007/s00701-023-05665-9 PMC10542718

[32] Schaller B, Baumann A (2003) Headache after removal of vestibular schwannoma via the retrosigmoid approach: a long-term follow-up-study. Otolaryngol Head Neck Surg 128(3):387–395 12646842 10.1067/mhn.2003.104

[33] Thomas M et al (2023) Premorbid psychological factors associated with long-term postoperative headache after microsurgery in vestibular schwannoma-a retrospective pilot study. Brain Sci. 10.3390/brainsci13081171 37626527 10.3390/brainsci13081171 PMC10452443

[34] Rutenkröger M et al (2024) Perceived Health Benefits in Vestibular Schwannoma Patients with Long-term Postoperative Headache: Insights from Personality Traits and Pain Coping – a cross-sectional study. J Pers Med 14(1):75 38248776 10.3390/jpm14010075 PMC10817612

[35] Lazak J et al (2024) Quality of life in patients after vestibular schwannoma surgery. Acta Neurochir (Wien) 166(1):33 38270649 10.1007/s00701-024-05936-z PMC10810939

[36] Organization, W.H. *Headache disorders*. 2024 2 March 2025]; Available from: https://www.who.int/news-room/fact-sheets/detail/headache-disorders.

[37] Ryzenman JM, Pensak ML, Tew JJ (2005) Headache: a quality of life analysis in a cohort of 1,657 patients undergoing acoustic neuroma surgery, results from the Acoustic Neuroma Association. Laryngoscope 115(4):703–711 15805885 10.1097/01.mlg.0000161331.83224.c5

[38] Bowers CA et al (2016) Surgical treatment of vestibular schwannoma: does age matter? World Neurosurg 96:58–65 27565466 10.1016/j.wneu.2016.08.054

[39] Lautenbacher S et al (2017) Age changes in pain perception: a systematic-review and meta-analysis of age effects on pain and tolerance thresholds. Neurosci Biobehav Rev 75:104–113 28159611 10.1016/j.neubiorev.2017.01.039

[40] Rigby PL et al (1997) Acoustic neuroma surgery: outcome analysis of patient-perceived disability. Am J Otol 18(4):427–435 9233481

[41] Machetanz K et al (2023) Sex differences in vestibular schwannoma. Cancers (Basel). 10.3390/cancers15174365 37686642 10.3390/cancers15174365 PMC10486905

[42] Mills SEE, Nicolson KP, Smith BH (2019) Chronic pain: a review of its epidemiology and associated factors in population-based studies. Br J Anaesth 123(2):e273–e283 31079836 10.1016/j.bja.2019.03.023 PMC6676152

[43] Brenner-Fliesser M, de Witt Huberts J, Wippert P-M (2018) Education, job position, income or multidimensional indices? Associations between different socioeconomic status indicators and chronic low back pain in a German sample: a longitudinal field study. BMJ Open 8:e020207 10.1136/bmjopen-2017-020207 PMC5931294 29705759

[44] Harner SG, Beatty CW, Ebersold MJ (1995) Impact of cranioplasty on headache after acoustic neuroma removal. Neurosurgery 36(6):1097–1100 7643987 10.1227/00006123-199506000-00005

[45] Sepehrnia A, Borghei-Razavi H (2015) Vestibular schwannoma between 1 and 3 cm: importance of the tumor size in surgical and functional outcome. Clin Neurol Neurosurg 129:21–26 25524483 10.1016/j.clineuro.2014.11.020

[46] Rimaaja T et al (2007) Headaches after acoustic neuroma surgery. Cephalalgia 27(10):1128–1135 17711494 10.1111/j.1468-2982.2007.01410.x

[47] Pruijn IMJ et al (2021) What determines quality of life in patients with vestibular schwannoma? Clin Otolaryngol 46(2):412–420 33326685 10.1111/coa.13691 PMC7986908

[48] Colloca L et al (2013) Placebo analgesia: psychological and neurobiological mechanisms. Pain 154(4):511–514 23473783 10.1016/j.pain.2013.02.002 PMC3626115

[49] Carlson ML et al (2015) What drives quality of life in patients with sporadic vestibular schwannoma? Laryngoscope 125(7):1697–1702 25546382 10.1002/lary.25110

[50] Moshtaghi O et al (2023) The effect of immediate microsurgical resection of vestibular schwannoma on hearing preservation. Otol Neurotol 44(6):600–604 37205868 10.1097/MAO.0000000000003893

[51] Jiramongkolchai P et al (2025) Hearing preservation outcomes in 230 consecutive patients with small vestibular schwannomas treated with microsurgery. Otol Neurotol 46(3):303–307 39794896 10.1097/MAO.0000000000004404

[52] Carlson ML et al (2023) Development and validation of a new disease-specific quality of life instrument for sporadic vestibular schwannoma: the Mayo Clinic Vestibular Schwannoma Quality of Life Index. J Neurosurg 138(4):981–991 36057121 10.3171/2022.7.JNS221104

[53] Bieńkowska K, Kostecka B, Kokoszka A (2025) Quality-of-life assessment instruments for patients with vestibular schwannoma: a systematic review. Braz J Otorhinolaryngol 91(3):101585 40120480 10.1016/j.bjorl.2025.101585 PMC11982967

